# Genome-Wide Identification and Characterization of Maize Long-Chain Acyl-CoA Synthetases and Their Expression Profiles in Different Tissues and in Response to Multiple Abiotic Stresses

**DOI:** 10.3390/genes15080983

**Published:** 2024-07-25

**Authors:** Zhenwei Yan, Jing Hou, Bingying Leng, Guoqi Yao, Changle Ma, Yue Sun, Qiantong Liu, Fajun Zhang, Chunhua Mu, Xia Liu

**Affiliations:** 1 Maize Research Institute, Shandong Academy of Agricultural Sciences, Jinan 250100, China; yanzwplant@sina.com (Z.Y.); lengbingying@saas.ac.cn (B.L.); yaoguoqi@saas.ac.cn (G.Y.); zhangfajun@saas.ac.cn (F.Z.); muchunhua@saas.ac.cn (C.M.); 2 School of Agriculture, Ludong University, Yantai 264001, China; houjing@m.ldu.edu.cn; 3 College of Life Sciences, Shandong Normal University, Jinan 250300, China; machangle@sdnu.edu.cn; 4 College of Agronomy, Qingdao Agricultural University, Qingdao 266109, China; sunyue3070601@163.com (Y.S.); 13356748152@163.com (Q.L.)

**Keywords:** long-chain acyl-CoA synthetase, genome-wide investigation, expression profiles, maize (*Zea mays* L.)

## Abstract

Long-chain acyl-CoA synthetases (LACSs) are essential enzymes that activate free fatty acids to fatty acyl-CoA thioesters, playing key roles in fatty acid (FA) catabolism, lipid synthesis and storage, epidermal wax synthesis, and stress tolerance. Despite their importance, comprehensive information about *LACS* genes in maize, a primary food crop, remains scarce. In the present work, eleven maize *LACS* genes were identified and mapped across five chromosomes. Three pairs of segmentally duplicated genes were detected in the maize *LACS* gene family, which underwent significant purifying selection (Ka/Ks < 1). Subsequently, phylogenetic analysis indicated that *ZmLACS* genes were divided into four subclasses, as supported by highly conserved motifs and gene structures. On the basis of the PlantCARE database, analysis of the *ZmLACS* promoter regions revealed various cis-regulatory elements related to tissue-specific expression, hormonal regulation, and abiotic stress response. RT-qPCR analysis showed that *ZmLACS* genes exhibit tissue-specific expression patterns and respond to diverse abiotic stresses including drought and salt, as well as phytohormone abscisic acid. Furthermore, using the STRING database, several proteins involved in fatty acid and complex lipid synthesis were identified to be the potential interaction partners of ZmLACS proteins, which was also confirmed by the yeast two-hybrid (Y2H) assay, enhancing our understanding of wax biosynthesis and regulatory mechanisms in response to abiotic stresses in maize. These findings provide a comprehensive understanding of *ZmLACS* genes and offer a theoretical foundation for future research on the biological functions of *LACS* genes in maize environmental adaptability.

## 1. Introduction

Long-chain acyl-CoA synthetases (LACSs, EC 6.2.1.3) are a subgroup of the carboxyl-CoA ligase superfamily (known as acyl-activating enzymes) that play crucial roles in fatty acid (FA) metabolism [1,2]. Before long-chain free FAs can be metabolized in cells, they must be activated into fatty acyl-CoA thioesters, a process catalyzed by LACS. During this reaction, free Fas are converted to an acyl-AMP intermediate, which then couples the activated carbon to the thiol group of CoA, releasing fatty acyl-CoA and AMP [3,4]. An AMP-binding domain and a conserved ACS signaling motif, both highly specific to the AMP-binding superfamily, are significant contributors to this activation process [5,6].

In higher plants, LACSs are located in various cellular organelles, including the chloroplast outer membrane, peroxisomes of germinating seeds, and the endoplasmic reticulum [7]. These enzymes are responsible for numerous processes, such as FA metabolism, lipid synthesis and storage, cutin polyester and cuticular wax synthesis, and stress tolerance. In *Arabidopsis thaliana*, a model plant organism, nine *AtLACS* genes have been identified, most of which are well-characterized [8]. For instance, *AtLACS1*, *AtLACS2*, *AtLACS4*, *AtLACS8*, and *AtLACS9* have redundant functions in epidermal lipid metabolism and are responsible for cutin and cuticular wax biosynthesis [9]. Studies on *AtLACS6* and *AtLACS7* have shown that these genes are situated in the peroxisome and exert complementary functions in FA β-oxidation in seeds [10]. Jessen et al. reported that *AtLACS1* and *AtLACS4* regulate the biosynthesis of pollen coat (tryphine)-specific lipids, thus contributing to plant viability [11]. Additionally, *AtLACS9*, located in plastids, works alongside *AtLACS1* in triacylglycerol biosynthesis [12]. *AtLACS3* is also associated with the cuticle formation pathway, with high expression in stem epidermal cells [13].

In addition to *Arabidopsis*, *LACS* genes have been identified in a variety of other plant species. In cotton (*Gossypium hirsutum*), GhLACS1, which shares high homology with AtLACS4/5, is crucial for normal microsporogenesis during early anther development [14]. In soybean (*Glycine max*), GmLACS2, located in the peroxisome, facilitates the decomposition of lipids and Fas during seed germination [15]. In castor (*Ricinus communis*), RcLACS2 selectively activates ricinoleate to its CoA thioester for triacylglycerol (TAG) biosynthesis [16]. In flax (*Linum usitatissimum*), LuLACS8 contributes to α-linolenic acid production in seed oil [17]. OsLACS9 in rice (*Oryza sativa*) is located in the chloroplast envelope, and its dysfunction leads to significant starch accumulation in the chloroplast and plant dwarfing [18]. BnLACS2 from rapeseed (*Brassica napus*), an *AtLACS2* orthologue, is located in the endoplasmic reticulum, mainly expressed in developing seeds, and responsible for seed oil production [19]. Furthermore, *HaLACS1* and *HaLACS2* in sunflower (*Helianthus annuus*), which exhibit sequence homology with *AtLACS8* and *AtLACS9*, respectively, play significant roles in oil synthesis in developing seeds [7]. Intriguingly, MdLACS2 in apple (*Malus pumila*) has been found to enhance plant resistance to drought and salt stress by mediating wax biosynthesis [20].

Maize (*Zea mays* L.) is not only a crucial food crop but also a major source of corn oil, widely used in the food industry and as a component in biodiesel [21,22]. Considering the essential roles of *LACS* genes in FA metabolism, lipid synthesis, storage, and resistance to abiotic stresses, it is imperative to conduct a genome-wide identification and characterization of *LACS* genes in maize. Herein, bioinformatic analysis was conducted on the maize *LACS* family. Our investigation included chromosomal distributions, gene duplication events, evolutionary relationships, gene structures, motif compositions, *cis*-regulatory elements, expression characteristics, and the protein–protein interaction network of all ZmLACS members. Collectively, our findings provide an extensive overview of *ZmLACS* genes and identify potential genes associated with tolerance to multiple abiotic stresses in maize.

## 2. Materials and Methods

### 2.1. Identification of LACS Genes in Maize

The sequences of nine Arabidopsis LACS proteins, retrieved from TAIR (https://www.arabidopsis.org/, accessed on 1 March 2024), were utilized as reference [8]. Subsequently, through employing default parameters and a significant e^−3^ value, a BLASTP search [23] was conducted in the maize genome sequences (B73 RefGen_v4), which were obtained from the Ensembl Plants database (https://plants.ensembl.org/index.html, accessed on 15 March 2024). To identify LACS proteins, the HMMER program (version 3.3.1) [24] was employed with the hidden Markov model (HMM) of the AMP-binding domain (PF00501), obtained from the Pfam database. Finally, the SMART database (http://smart.embl-heidelberg.de, accessed on 17 March 2024) [25], the NCBI Conserved Domain Database (https://www.ncbi.nlm.nih.gov/cdd/, accessed on 18 March 2024) [26], and the Pfam database (http://pfam.xfam.org/search, accessed on 19 March 2024) [27] were utilized to verify the gene sequences and identify the members of the maize *LACS* family.

### 2.2. Bioinformatics Analysis of ZmLACS Genes

Various parameters, such as the number of amino acid (AA) residues, GRAVY, pI, and MW, were determined through the ExPASY webtool (https://web.expasy.org/protparam/, accessed on 3 April 2024).

### 2.3. Characterization of Chromosomal Locations and Gene Duplication

Detailed information on the chromosomal locations of all *ZmLACS* genes was acquired based on the maize genome database (www.maizegdb.org, accessed on 10 April 2024). MapChart was employed to construct the chromosomal location map of *ZmLACS* genes and the relative distances [28].

For the syntenic analysis of *ZmLACS* genes, MCScanX was employed to identify both tandem and segmental duplications [29]. The obtained data were visualized based on the Advanced Circos module in TBtools [30]. For the co-linearity analysis of *LACS* genes between maize and Arabidopsis, the dual synteny plotter module in TBtools was used. To assess the selection pressure on *ZmLACS* genes, key parameters such as Ks (synonymous), Ka (non-synonymous), and the Ka/Ks ratio were calculated. This analysis, which provides insights into evolutionary dynamics and selection pressures, was conducted using TBtools software v1.098 (https://github.com/CJ-Chen/TBtools, accessed on 12 April 2024) [31].

### 2.4. Multiple Sequence Alignment and Construction of the Phylogenetic Tree

Multiple sequence alignment was conducted on LACS protein sequences from Arabidopsis and maize using MUSCLE software (v3.6) [32]. Meanwhile, a neighbor-joining (NJ) tree of LACS proteins was established using the MEGA_X_10.1.7 with bootstrap analysis employing 1000 iterations and default parameters [33].

### 2.5. Conserved Motifs and Gene Structure Analysis

The conserved motifs of ZmLACS proteins were estimated with MEME v5.4.1 (http://meme-suite.org/tools/meme, v5.4.1, accessed on 14 April 2024), with ten motifs to be identified and obtained [34]. The GSDS2.0 (http://gsds.gao-lab.org/, accessed on 15 April 2024) program was employed to elucidate the gene structures of *ZmLACS*, which are composed of untranslated region (UTR) composition, introns, and coding sequences (exons) [35].

### 2.6. Cis-Acting Element Analysis of ZmLACSs

To determine the putative *cis*-acting elements of *ZmLACS* genes, 1.5 kb genome sequences upstream of the initiation codon (ATG) were retrieved from the PlantCARE database (http://bioinformatics.psb.ugent.be/webtools/plantcare/html/, accessed on 25 April 2024) [36], and the resulting graph was constructed using the GSDS2.0 (http://gsds.gao-lab.org/, accessed on 25 April 2024) program [35]. Eight representative *cis*-elements were selected, including G-box (light responsiveness), ABRE (abscisic acid responsiveness), CGTCA-motif (MeJA responsiveness), TCA-element (salicylic acid responsiveness), LTR (low-temperature responsiveness), ARE (anaerobic induction), CAT-box (Meristem expression), and GCN4_motif (endosperm expression).

### 2.7. Protein–Protein Interaction Network

The Search Tool for the Retrieval of Interaction Gene/Proteins (STRING, https://string-db.org/, accessed on 7 May 2024) contains comprehensive information about interaction relationships between known and hypothetical proteins/genes. Here, the PPI network was established through the STRING database, and the network diagram was drawn using Cytoscape v3.6.0 (USA) [37].

### 2.8. Y2H Assay

The full-length coding sequences of maize palmitoyl-acyl carrier protein thioesterase 1 (ZmPAT1), maize palmitoyl-acyl carrier protein thioesterase 2 (ZmPAT2), maize palmitoyl-acyl carrier protein thioesterase 3 (ZmPAT3), maize very-long-chain aldehyde decarbonylase 1-4 (ZmVLCAD1-4), and maize very-long-chain aldehyde decarbonylase 1-5 (ZmVLCAD1-5) were amplified and inserted into pGBKT7 vector, and ZmLACS1, ZmLACS2, ZmLACS4.1, ZmLACS4.2, ZmLACS6.2, ZmLACS8.1, and ZmLACS9.2 were cloned into pGADT7 vector. The yeast strain Y2HGold was utilized to carry out the assay in adherence to the guidelines specified by Clontech. Both the resulting constructs and their corresponding empty vectors were co-transformed into this strain. The specific primers used for the Y2H assay have been itemized within Table S1.

### 2.9. Multiple Stress Treatments

Maize seed (B73) was grown in an accurately controlled growth chamber (Percival). The chamber maintained a 16-h photoperiod with a temperature, light intensity, and relative humidity of 25 °C, 400 µmol m^−2^ s^−1^, and 70%, respectively. Subsequently, maize seedlings were cultured in half-strength Hoagland’s solution (Coolaber, Beijing, China) until the three-leaf stage and then were transferred to 1/2 Hoagland solution with or without 10% PEG 6000, 200 mM NaCl, and 50 µM ABA specific durations.

### 2.10. RNA Isolation and Quantitative RT-PCR Analysis

Following accurate protocols with the FastPure^®^ Universal Plant Total RNA Isolation Kit (Vazyme, Nanjing, China), total RNA extraction was conducted following the below steps. 1. Tissue Preparation: The first step typically involves collecting the plant tissue and grinding it into a fine powder using liquid nitrogen to prevent the degradation of RNA. 2. Lysis: The ground tissue is then lysed using a lysis buffer that contains high concentrations of detergents like guanidinium thiocyanate or CTAB to lyse cells and solubilize cellular components. This step is crucial for breaking down the cell walls and releasing the intracellular contents. 3. Phase Separation: After lysis, the mixture undergoes phase separation using an organic solvent such as phenol–chloroform–isoamyl alcohol (PCI) mixture. This step helps in separating the RNA from proteins, lipids, and other contaminants. 4. RNA Precipitation: The RNA is then precipitated by adding a precipitating agent like lithium chloride (LiCl) followed by ethanol to form a pellet. This step is essential for concentrating the RNA. 5. Washing and Resuspension: The RNA pellet is washed to remove any residual salts and organic solvents. Subsequently, the RNA is resuspended in a suitable buffer for further use. 6. Quality and Quantity Assessment: Finally, the isolated RNA is assessed for its quality (integrity number using an agarose gel electrophoresis) and quantity (using a spectrophotometer) to ensure it is suitable for downstream applications such as RT-PCR, microarray analysis, or next-generation sequencing.

Subsequently, total RNA (1 µg) was reversely-transcripted using the HiScript^®^ III RT SuperMix for qPCR(+gDNA-wiper) kit (Vazyme). RT-qPCR was performed using the ChamQ Universal SYBR-qPCR Master Mix (Vazyme) on a Stratagene Mx3000P real-time system cycler (Agilent, Santa Clara, CA, USA) following the below protocols. 1. Preparation of Reagents: Before starting the qPCR reaction, ensure that all reagents are properly prepared and stored according to the manufacturer’s instructions. This typically includes preparing a master mix that contains necessary components such as dNTPs, MgCl_2_, buffer, and SYBR Green I dye. 2. Setting Up the Reaction: The reaction setup generally involves adding multiple components (template DNA, forward and reverse primers, SYBR Green I dye master mix, and nuclease-free water) into a PCR tube. 3. PCR Cycler Settings: Program the thermal cycler according to the manufacturer’s recommendations. Typically, this includes an initial denaturation step (to separate DNA strands), followed by a series of amplification cycles consisting of denaturation, annealing (primer binding), and extension (DNA synthesis). The exact settings can vary depending on the target DNA and the desired product size. 4. Fluorescence Monitoring: During the amplification cycles, the fluorescence of the SYBR Green I dye increases as more DNA is amplified. This increase is detected by the real-time PCR system and recorded throughout the reaction. 5. Data Analysis: After the PCR cycle, analyze the data to determine the presence and quantity of the target DNA. The threshold cycle (Ct) value indicates the number of cycles required for the fluorescence signal to exceed a certain threshold, which correlates with the initial amount of target DNA in the sample. 6. Quality Control: It is important to perform quality control checks, such as including negative controls (without template DNA) and positive controls (with known amounts of target DNA) to ensure the accuracy and reliability of the results.

In addition, actin1 (GRMZM2G126010) was chosen as the reference control for the analysis. Each experiment included 3 technical replicates, and 3 independent biological experiments were conducted to validate the results. The primer pairs utilized for RT-qPCR assay are detailed in Table S1.

### 2.11. Statistical Analysis

Statistical tests of datasets comprising two distinct groups were performed using Student’s *t*-test. “ns” denotes no obvious difference between the control and experimental groups. Significant differences were indicated by *, **, ***, and ****, representing *p* < 0.05, *p* < 0.01, *p* < 0.001, and *p* < 0.0001, respectively. All values were reported as means ± SD.

## 3. Results

### 3.1. Identification and Sequence Analysis of ZmLACS Genes in Maize

By employing the AA sequences of Arabidopsis LACSs (AtLACS1–AtLACS9), the hidden Markov model (HMM) [24] and BlastP algorithm search [23] were conducted against the maize genome. Subsequently, SMART [25], CDD [26], and Pfam databases [27] were utilized to verify the existence of an AMP-conserved domain in *LACS* genes.

This comprehensive method led to the identification of 11 putative maize *LACS* genes. These genes were named *ZmLACS1*, *ZmLACS2*, *ZmLACS4.1*, *ZmLACS4.2*, *ZmLACS4.3*, *ZmLACS6.1*, *ZmLACS6.2*, *ZmLACS8.1*, *ZmLACS8.2*, *ZmLACS9.1*, and *ZmLACS9.2*, according to their homolog sequences in Arabidopsis. The names *ZmLACS4.1*, *ZmLACS4.2*, and *ZmLACS4.3* were chosen instead of *ZmLACS3* due to their highest similarity to the *AtLACS4* gene. *ZmLACS6.1* and *ZmLACS6.2*, *ZmLACS8.1* and *ZmLACS8.2*, and *ZmLACS9.1* and *ZmLACS9.2* were named due to their closer relationships with Arabidopsis *LACS* genes. Moreover, the genomic locations, open-reading-frame (ORF) lengths, AA numbers, grand averages of hydropathy (GRAVY), iso-electric points (PI), and molecular weights (MW) of all *ZmLACS* genes were systematically characterized (Table S2). The *ZmLACS* gene family exhibited similar ORF lengths, ranging from 1965 bp (*ZmLACS4.1*) to 2178 bp (*ZmLACS8.1*/*ZmLACS8.2*). Correspondingly, AA numbers ranged from 655 to 726. The estimated MWs of 11 ZmLACS proteins ranged from 72.67 kDa (ZmLACS4.1) to 80.10 kDa (ZmLACS8.2). Five members had a pI above 7, with the highest being 8.69 (ZmLACS9.1), while the remaining ZmLACS proteins had a pI below 7, with the lowest being 5.68 (ZmLACS4.1). Additionally, all 11 ZmLACS proteins were predicted to be hydrophilic, with GRAVY values below zero.

A conserved acyl-CoA synthetase (ACS) signaling motif and an AMP-binding domain, both highly conserved domains, have been reported to be present in acyl-activating enzymes [5,6]. Based on AA sequences, a multiple alignment of maize and Arabidopsis LACSs was conducted. As depicted in Figures S1 and S2, the AA sequences of the 9 AtLACS proteins and the 11 ZmLACS proteins exhibit high homology and conservation, with nearly all ZmLACS proteins containing these two conserved structural domains.

### 3.2. Chromosomal Localization and Syntenic Analysis of ZmLACS Genes

**Figure 1:**
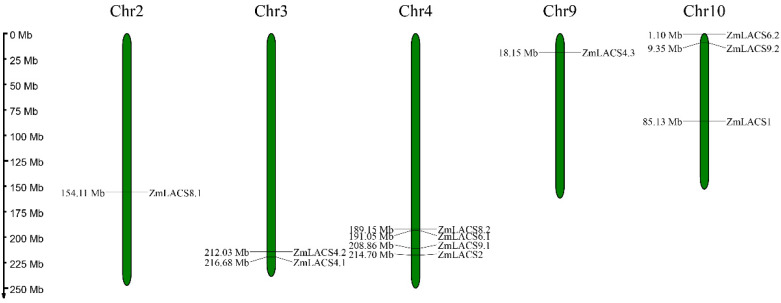
Chromosomal locations of *LACS* genes in maize. Eleven *ZmLACS* genes were distributed across chromosomes 2, 3, 4, 9, and 10. The corresponding chromosome numbers are illustrated at the uppermost section of each chromosome.

Using MapChart software (v2.0), the 11 *ZmLACS* genes were mapped onto five chromosomes. As displayed in Figure 1, most *ZmLACS* members (*ZmLACS2*, *ZmLACS6.1*, *ZmLACS8.2*, and *ZmLACS9.2*) were distributed on chromosome 4, and chromosome 10 contained three *ZmLACSs* (*ZmLACS1*, *ZmLACS6.2*, and *ZmLACS9.2*). In addition, *ZmLACS4.1* and *ZmLACS4.2* were located on chromosome 3, with chromosomes 2 and 9 each featuring one *ZmLACS*.

**Figure 2:**
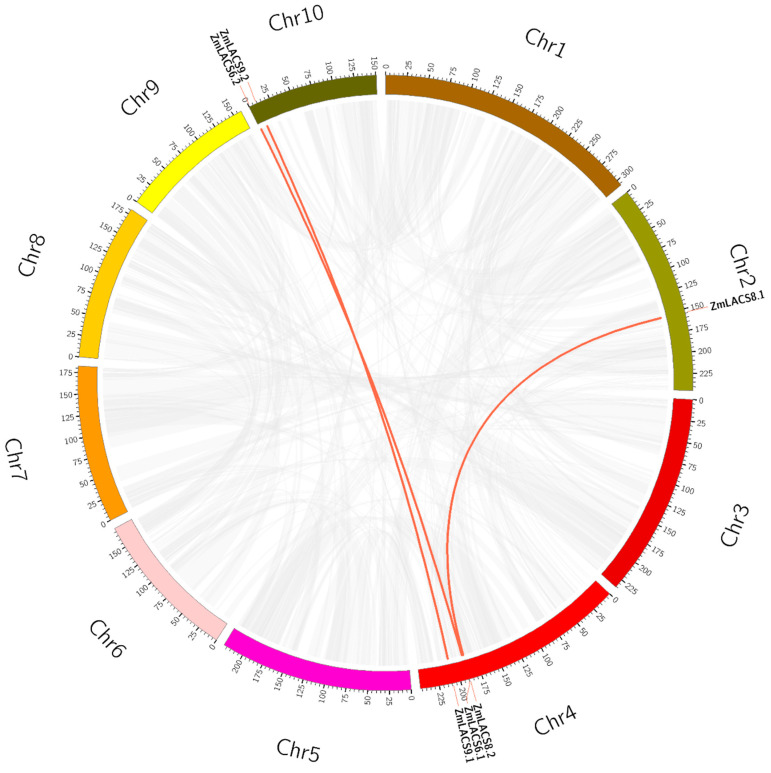
Synteny relationships of the *ZmLACS* gene family. The red lines in the circle represent the duplicated *LACS* gene pairs in the maize. The gray lines illustrate gene collinearity regions in the maize genome. The outer circle segments represent individual maize chromosomes. Scale bars on chromosomes provide a visual reference for chromosomal lengths in megabases (Mb).

Considering the crucial function of duplication events in the expansion of gene families during evolution, a syntenic analysis was performed within the maize genome [38,39]. Based on sequence identity and query coverage, three gene pairs (*ZmLACS6.1*/*ZmLACS6.2*, *ZmLACS8.1*/*ZmLACS8.2*, and *ZmLACS9.1*/*ZmLACS9.2*) were identified as segmentally duplicated and were situated on chromosomes 2, 4, and 10 (Figure 2). Interestingly, *ZmLACS1*, *ZmLACS2*, *ZmLACS4.1*, *ZmLACS4.2*, and *ZmLACS4.3* were not involved in duplication events. Additionally, no tandem duplication was found for the *ZmLACS* genes in maize. These findings suggest that segmental duplication events, rather than tandem repeat events, drive the expansion of ZmLACS proteins.

To identify the evolutionary features of *ZmLACS* genes, we determined the Ka and Ks of duplicated gene pairs (Table S3). The Ka/Ks values of the three gene pairs mentioned above ranged between 0.14 and 0.16, all below 1, indicating that these *ZmLACS* genes have undergone significant purifying selection (Ka/Ks < 1) [40]. Intriguingly, no orthologous gene pair was identified when comparing the genome-wide data of maize LACSs with those of Arabidopsis (Figure S3), which may suggest a relatively distant genetic divergence between maize and Arabidopsis.

### 3.3. Phylogenetic Analysis of LACS Genes in Maize

**Figure 3:**
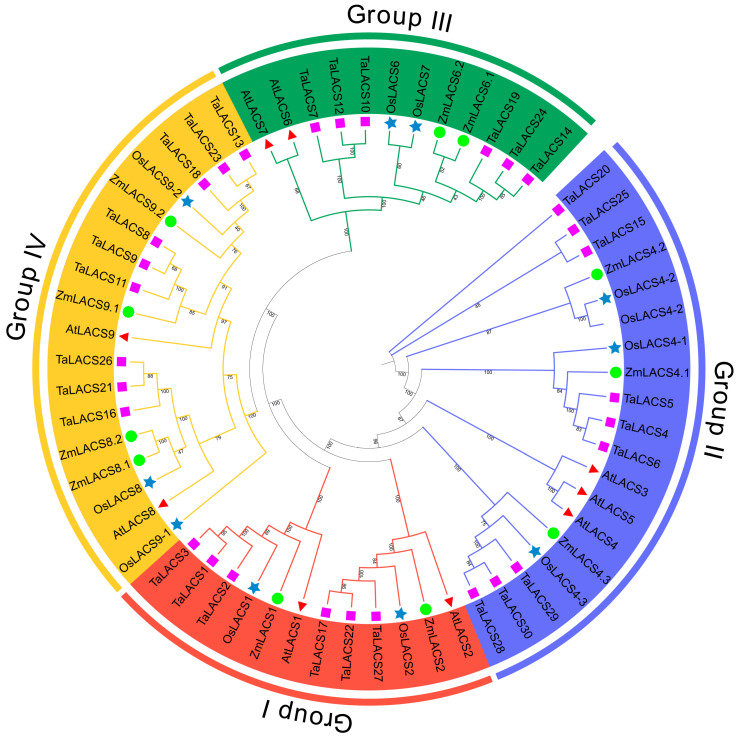
Phylogenetic analysis and classification of LACS proteins in maize, Arabidopsis, wheat, and rice. The phylogenetic tree, constructed with 1000 bootstrap replicates, was visually drawn by categorizing it into distinct subgroups. Each subgroup, denoted by different colors, was further labeled as I–IV, representing the four identified subgroups. Arrowheads represent LACS proteins in Arabidopsis, circles indicate LACS proteins in maize, square frames represent LACS proteins in wheat, and five-pointed stars indicate LACS proteins in rice.

To explore the evolutionary relationships among the 11 ZmLACS proteins, a phylogenetic tree was established using MEGA7 software (version 7.0.21) according to protein sequences via the NJ approach. In total, 61 LACS protein sequences from maize and three additional species (Arabidopsis, wheat, and rice) were used [41,42]. As illustrated in Figure 3, interestingly, 11 ZmLACS proteins were assigned into four subgroups (I to IV). Two ZmLACS members belong to group I or III, group II contains three ZmLACS members, and group IV contains four ZmLACS members. Furthermore, a comprehensive analysis of the phylogenetic tree and duplication events revealed that duplicated gene pairs exhibit closer evolutionary relationships.

### 3.4. Conserved Motifs and Gene Structures of the ZmLACS Genes

**Figure 4:**
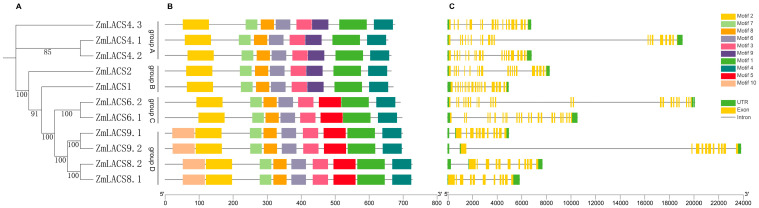
Phylogenetic relationship, conserved motifs, and gene structure of maize LACSs: (**A**) The neighbor-joining (NJ) phylogenetic tree of maize LACS proteins. (**B**) Conserved motifs distributions of 11 ZmLACS proteins. Diverse colored boxes illustrate conserved motifs (1–10). (**C**) Gene structure of maize LACSs. The yellow box represents the coding region (CDS), the green box represents the untranslated region (UTR), and the black line represents the intron.

To further examine the evolutionary conservation of all *LACS* genes in maize, an independent NJ tree was constructed according to the protein sequences of all maize LACSs, and the correspondingly conserved motifs were analyzed using MEME online software (v 5.1.0). As shown in Figure S4, ten conserved motifs (Motif 1–10) were identified. Meanwhile, through checking the multiple alignment of maize and Arabidopsis LACSs (Figure S1), we also found that motif 7 contains the AMP-binding domain, and the conserved ACS signaling motif is located in motif 1. According to the phylogenetic analysis and the distribution of motifs, ZmLACSs were grouped into four clusters, with similar motif compositions shared by ZmLACS proteins within the same cluster (Figure 4A,B). For instance, Motif 9 was specifically detected in ZmLACSs of groups A and B, while Motif 5 was present in 6 *ZmLACS* genes of groups C and D. In addition, Motif 10 was found in four members (*ZmLACS8.1*, *ZmLACS8.2*, *ZmLACS9.1*, and *ZmLACS9.2*) of group D.

In addition to the composition of conserved motifs, gene structure also contributed to the evolutionary process of *ZmLACS* genes [43]. To further examine the features of *ZmLACSs*, we assessed the gene structure of 11 *ZmLACSs* using GSDS software (v2.0). As shown in Figure 4C, excluding the size of the introns, the *ZmLACS* genes within the same group displayed similar exon-intron structures. For instance, subclades A and B both contained 19 exons, while subclade C had two members with 23 exons each, and subclade D had four *ZmLACS* genes with 11 exons each. Collectively, these results indicate that the composition of conserved motifs and gene structure are involved in the evolutionary divergence and functional differences of maize LACS proteins.

### 3.5. Cis-Element Analysis in the Promoter Regions of ZmLACS Genes in Maize

**Figure 5:**
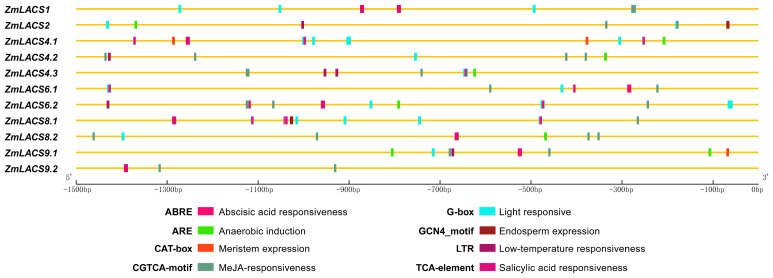
*Cis*-elements analysis in *ZmLACS* promoter regions. Distinct colored boxes at the bottom represent diverse *cis*-elements.

Gene expression diversity under various conditions is closely correlated with the promoter region [44]. To investigate potential *cis*-elements in the promoter regions of 11 *ZmLACS* genes, we retrieved and analyzed 1.5 kb upstream sequences from the start codon (ATG) for each *ZmLACS* gene. A total of 18 types of *cis*-elements related to light response, plant hormone response, abiotic stress, tissue-specific expression, and MYB binding were identified (Figure 5 and Table S4). Notably, hormone-responsive elements (abscisic acid, methyl jasmonate, salicylic acid, and auxin), light-responsive elements, and low-temperature-responsive elements were the most enriched. This suggests potential roles for ZmLACS proteins in response to various abiotic stresses and hormonal regulation. Additionally, circadian rhythm response elements (circadian), cell cycle regulation elements (MSA-like), and tissue-specific regulatory elements (CAT-box and o2-site motif) were also found in *ZmLACS* promoter regions, highlighting the diverse functions of the *ZmLACS* gene family in maize growth and development.

### 3.6. Expression Analysis of Maize LACSs in Multiple Tissues and Developmental Stages

To preliminarily elucidate the expression profiles of *ZmLACS* genes in diverse tissues and developmental stages, we conducted RT-qPCR to explore the expression data of all *ZmLACS* genes in the primary root (V1), stem and SAM (V1), pooled leaves (V1), first internode (V5), immature tassel (V13), meiotic tassel (V18), silks (R1), anthers (R1), pericarp 18 DAP, endosperm 22 DAP, embryo 22 DAP, seed 2, 4, 6, 8, 16, and 20 DAP.

**Figure 6:**
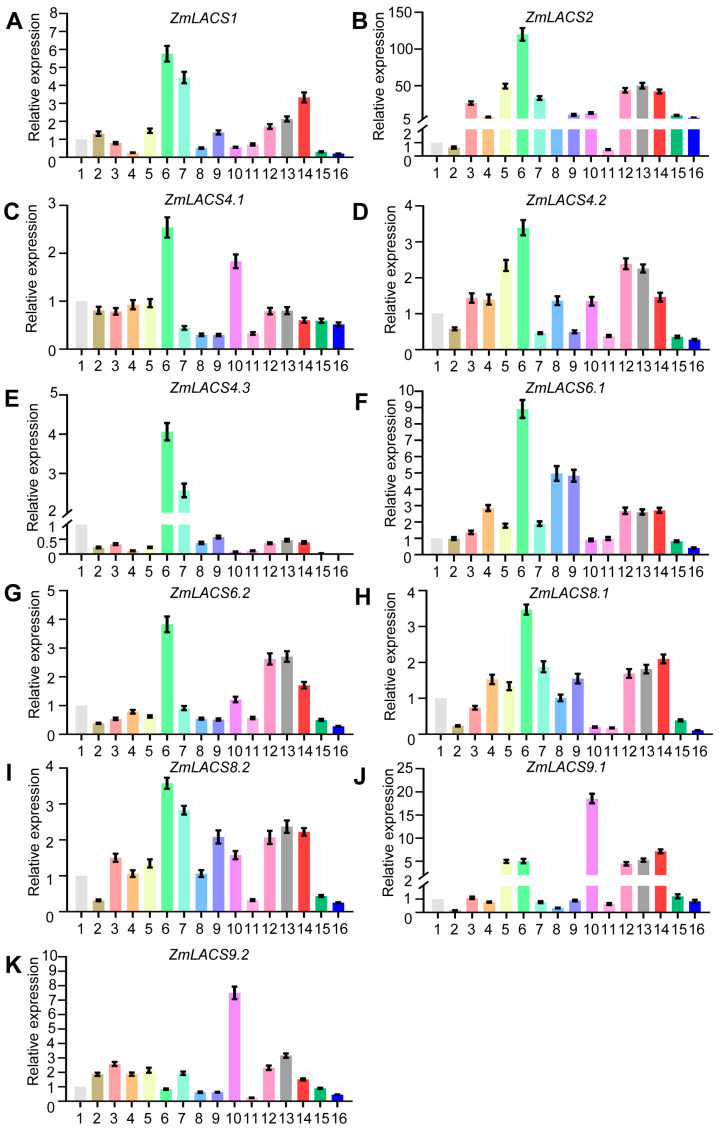
Relative expression analysis of *ZmLACS* genes in different tissues. The expression levels of *ZmLACS1* (**A**), *ZmLACS2* (**B**), *ZmLACS 4.1* (**C**), *ZmLACS4.2* (**D**), *ZmLACS4.3* (**E**), *ZmLACS6.1*(**F**), *ZmLACS6.2* (**G**), *ZmLACS8.1* (**H**), *ZmLACS8.2* (**I**), *ZmLACS9.1* (**J**), and *ZmLACS9.2* (**K**) in primary root (V1) (1), pooled leaves (V1) (2), stem and SAM (V1) (3), first internode (V5) (4), immature tassel (V13) (5), meiotic tassel (V18) (6), silks (R1) (7), anthers (R1) (8), pericarp 18 DAP (9), embryo 22 DAP (10), endosperm 22 DAP (11), seed 2 DAP (12), seed 4 DAP (13), seed 8 DAP (14), seed 16 DAP (15), and seed 20 DAP (16) using RT-qPCR analysis. Expression in primary root (V1) was set to 1.00. Data shown are means ± SD of three biological replicates.

As shown in Figure 6, all *ZmLACS* genes, except for *ZmLACS9.1* and *ZmLACS9.2*, exhibited the highest expression in the meiotic tassel (V18), suggesting that FA metabolism is highly active in this tissue. *ZmLACS9.1* and *ZmLACS9.2*, two duplicated genes from group A, showed the highest expression in the embryo at 22 DAP and had similar expression profiles across multiple tissues and stages, indicating potential functional redundancy and key roles in maize embryo development. Another interesting observation was the expression pattern during different seed developmental stages (days after pollination, DAP). All *ZmLACS* genes displayed higher expression levels at seed 2, 4, and 8 DAP, which then declined at seed 16 and 20 DAP. This pattern suggests that these genes are involved in the early accumulation of FAs in maize seeds. Additionally, besides the meiotic tassel (V18), embryo 22 DAP, seed 2, 4, and 8 DAP, *ZmLACS1*, *ZmLACS4.3*, *ZmLACS8.1*, and *ZmLACS8.2* were highly expressed in silks (R1), while *ZmLACS2*, *ZmLACS4.2*, and *ZmLACS9.1* were highly expressed in immature tassel (V13).

### 3.7. Expression Profiles of ZmLACSs under Various Stress Conditions

**Figure 7:**
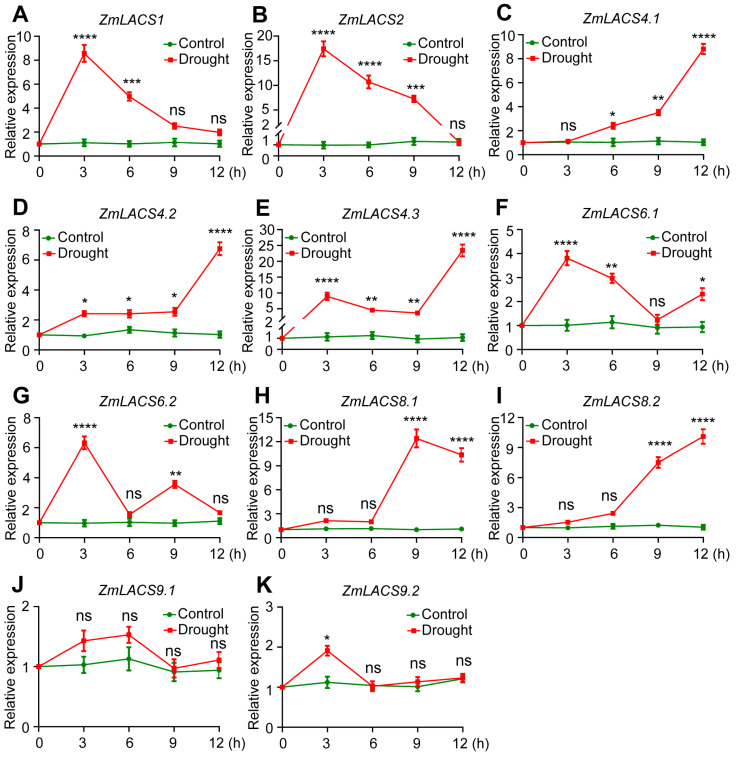
Relative expression of *ZmLACS* genes under drought treatment. The expression levels of *ZmLACS1* (**A**), *ZmLACS2* (**B**), *ZmLACS 4.1* (**C**), *ZmLACS4.2* (**D**), *ZmLACS4.3* (**E**), *ZmLACS6.1* (**F**), *ZmLACS6.2* (**G**), *ZmLACS8.1* (**H**), *ZmLACS8.2* (**I**), *ZmLACS9.1* (**J**), and *ZmLACS9.2* (**K**) using RT-qPCR analysis. Expression in control was set to 1.00. Data shown are means ± SD of three biological replicates. ns indicates no significant difference to the corresponding controls. *, **, ***, and **** represent *p* < 0.05, *p* < 0.01, *p* < 0.001, and *p* < 0.0001 vs. control, respectively (Student’s *t*-test).

**Figure 8:**
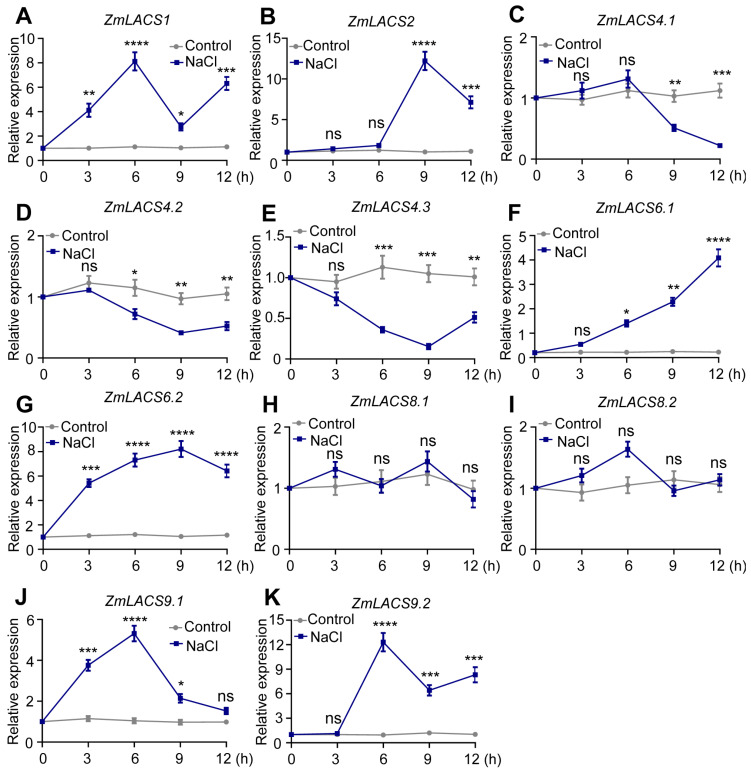
Relative expression of *ZmLACS* genes under NaCl treatment. The expression levels of *ZmLACS1* (**A**), *ZmLACS2* (**B**), *ZmLACS 4.1* (**C**), *ZmLACS4.2* (**D**), *ZmLACS4.3* (**E**), *ZmLACS6.1* (**F**), *ZmLACS6.2* (**G**), *ZmLACS8.1* (**H**), *ZmLACS8.2* (**I**), *ZmLACS9.1* (**J**), and *ZmLACS9.2* (**K**) using RT-qPCR analysis. Expression in control was set to 1.00. Data shown are means ± SD of three biological replicates. ns indicates no significant difference to the corresponding controls. *, **, ***, and **** represent *p* < 0.05, *p* < 0.01, *p* < 0.001, and *p* < 0.0001 vs. control, respectively (Student’s *t*-test).

**Figure 9:**
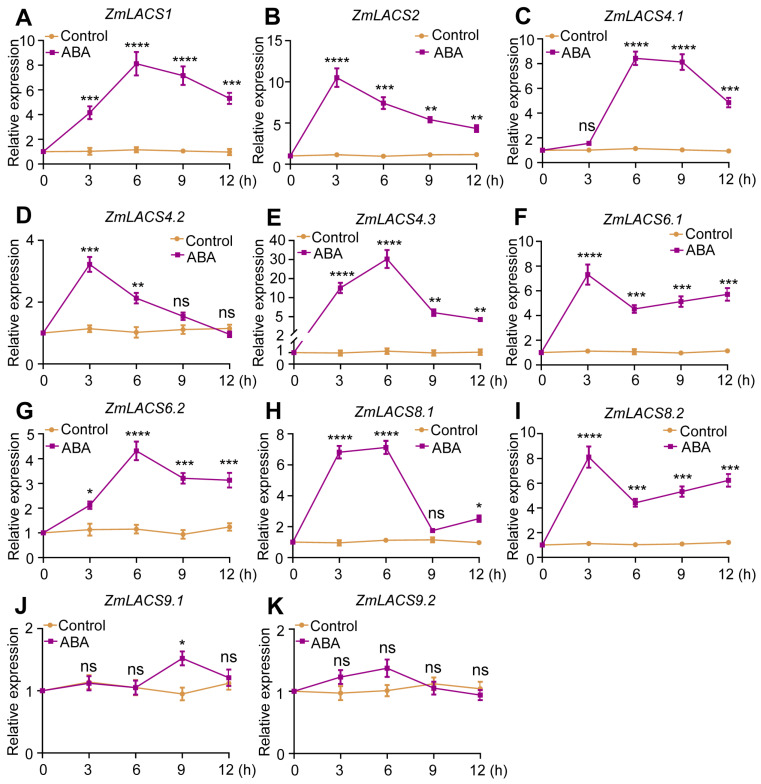
Relative expression of *ZmLACS* genes under ABA treatment. The expression levels of *ZmLACS1* (**A**), *ZmLACS2* (**B**), *ZmLACS 4.1* (**C**), *ZmLACS4.2* (**D**), *ZmLACS4.3* (**E**), *ZmLACS6.1* (**F**), *ZmLACS6.2* (**G**), *ZmLACS8.1* (**H**), *ZmLACS8.2* (**I**), *ZmLACS9.1* (**J**), and *ZmLACS9.2* (**K**) using RT-qPCR analysis. Expression in control was set to 1.00. Data shown are means ± SD of three biological replicates. ns indicates no significant difference to the corresponding controls. *, **, ***, and **** represent *p* < 0.05, *p* < 0.01, *p* < 0.001, and *p* < 0.0001 vs. control, respectively (Student’s *t*-test).

To determine the potential roles of ZmLACSs in multiple abiotic stresses and hormone induction, we examined the transcriptional alterations of *ZmLACS* genes exposed to drought (PEG6000), salt (NaCl), and abscisic acid (ABA) (Figure 7, Figure 8 and Figure 9). The data indicated that the majority of *ZmLACS* genes reacted to various treatments.

Under drought conditions, the transcript abundances of *ZmLACS1*, *ZmLACS2*, *ZmLACS6.1*, and *ZmLACS6.2* rapidly peaked after 3 h of treatment and then gradually reduced. On the contrary, the expression levels of *ZmLACS4.1*, *ZmLACS4.2*, *ZmLACS4.3*, *ZmLACS8.1*, and *ZmLACS8.2* were remarkably elevated during the late stages of drought treatment. Interestingly, the transcript abundances of *ZmLACS9.1* and *ZmLACS9.2* remained almost unchanged in response to drought. Similarly, most *ZmLACS* genes, except for *ZmLACS8.1* and *ZmLACS8.2*, markedly responded to salt stress. For example, salt stress dramatically induced the transcriptional upregulation of *ZmLACS1*, *ZmLACS9.1*, and *ZmLACS9.2* at the early stage. However, significant inductions of *ZmLACS2*, *ZmLACS6.1*, and *ZmLACS6.2* were detected at the later stages. Additionally, salt stress remarkably decreased the expression levels of *ZmLACS4.1*, *ZmLACS4.2*, and *ZmLACS4.3*.

As expected, ABA treatment did not result in obvious changes in the expression of *ZmLACS9.1* and *ZmLACS9.2*. However, the remaining *ZmLACS* genes rapidly attained their peak expression levels within 3–6 h following ABA treatment.

### 3.8. Protein Interaction Network Analysis of ZmLACS Proteins

**Figure 10:**
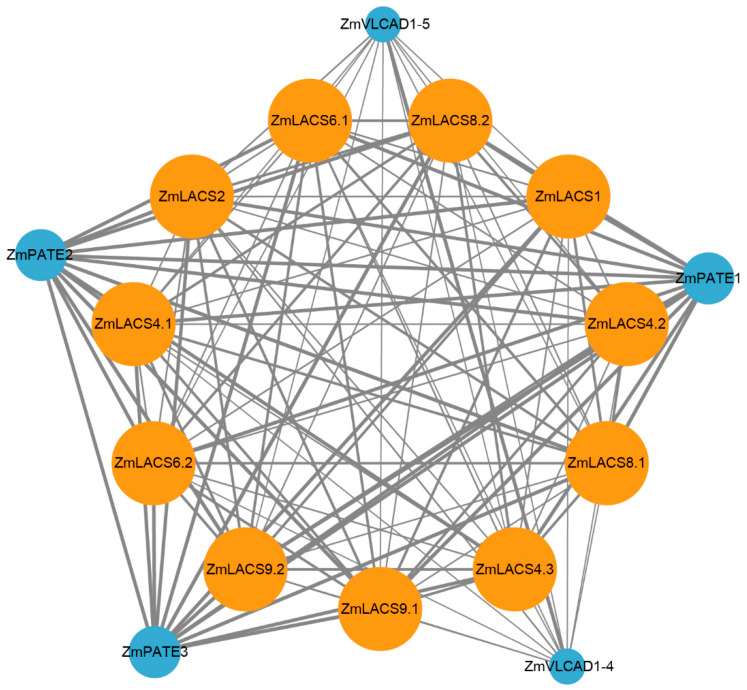
Predicted protein–protein interaction networks (PPIs) using the STRING tool. Within the PPI network, every node encapsulates all proteins derived from the corresponding single gene. The size of each node indicates the degree of interaction, while the thickness of the edges signifies the strength of protein–protein interactions. Nodes representing ZmLACSs are drawn in yellow, while proteins interacting with ZmLACSs are highlighted in blue.

**Figure 11:**
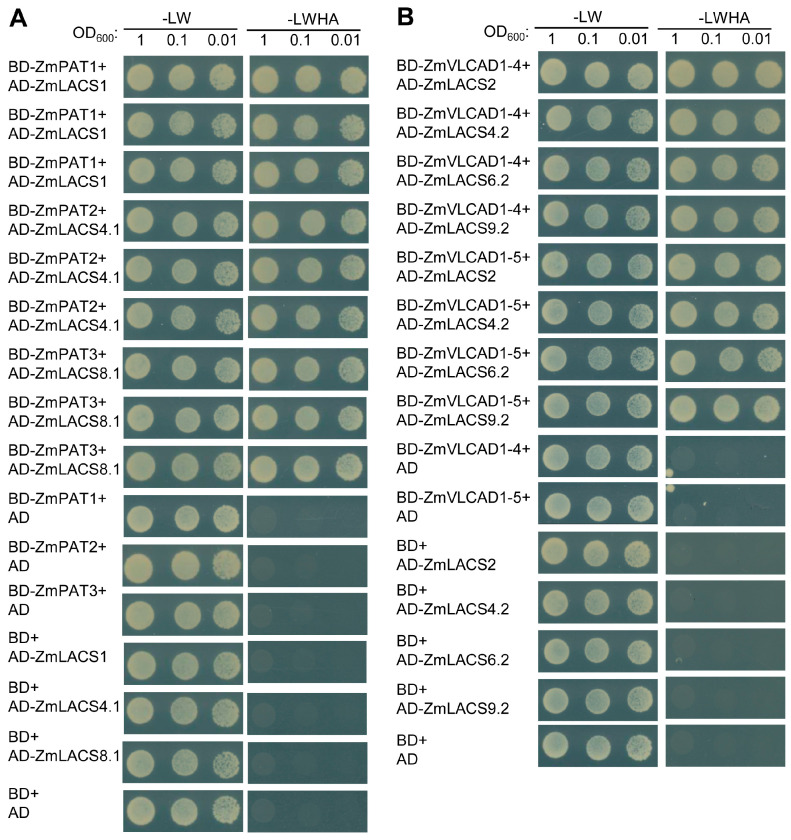
(**A**) Y2H assay showing the interaction between ZmPAT1/2/3 and ZmLACS1/4.1/8.1. (**B**) Y2H assay showing the interaction between ZmVLCAD1-4/1-5 and ZmLACS2/4.2/6.2/9.2. Yeast cells were grown on selection plates: SD-LW(-Leu-Trp) and SD-LWHA(-Leu-Trp-His-Ade). BD: pGBKT7, AD: pGADT7.

To further elucidate the potential biological functions and regulatory networks of ZmLACS proteins, we constructed a protein interaction network based on *Arabidopsis* orthologous proteins using the STRING database. As illustrated in Figure 10 and Table S5, several proteins related to complex lipid and FA synthesis showed notable interactions with ZmLACS proteins. For example, three palmitoyl-acyl carrier protein thioesterases (ZmPATE1, ZmPATE2, and ZmPATE3) function in plastidial FA biosynthesis and modulate C16:0 FA accumulation [45,46]. Moreover, two very-long-chain aldehyde decarboxylases (ZmVLCAD1-4 and ZmVLCAD1-5) convert aldehydes to alkanes and modulate the biosynthesis of very-long-chain FAs (e.g., polyesters) in pollen exine, anther tapetum, and cuticles [47]. In addition, ZmPAT1/2/3, ZmVLCAD1-4/1-5, and ZmLACS1/2/4.1/4.2/6.2/8.1/9.2 were selected for further validation. As shown in Figure 11, the interactions between ZmPAT1/2/3 and ZmLACS1/4.1/8.1 and the ZmVLCAD1-4/1-5-ZmLACS2/4.2/6.2/9.2 interactions were verified using a yeast-two hybrid assay. Collectively, these findings further support the view that ZmLACS proteins are crucial in FA catabolism and lipid synthesis.

## 4. Discussion

LACSs, which convert free FAs into fatty acyl-CoA thioesters, play crucial roles in FA catabolism, lipid synthesis and storage, epidermal wax synthesis, and stress tolerance. Although *LACS* gene families have been well characterized in multiple species, detailed information about this family in maize is lacking. In this study, 11 *LACSs* were identified in maize, with a genome size of 2106 Mb [48]. Additionally, 9, 11, 30, 11, 34, 22, 20, 12, and 11 *LACS* genes were found in Arabidopsis, apple (*Malus* × *domestica*), wheat, pecan (*Carya illinoinensis*), *Brassica napus*, cotton (*G. hirsutum*), cotton (*G. barbadense*), cotton (*G. raimondii*), and cotton (*G. arboreum*), respectively, with genome sizes of 157, 742, 17,000, 690, 840, 2300, 2220, 885, and 1746 Mb, respectively [49,50,51,52,53]. Interestingly, our attention was drawn to the phenomenon that genome size may not be positively correlated with the number of *LACS* genes. Further studies are needed to investigate this observation.

Conserved motifs have been reported to be associated with multiple biological processes, including subcellular localization, protein interactive ability, and transcriptional regulation [54]. Herein, motifs 1–10 were identified in 11 ZmLACS proteins. Notably, the conserved motifs of the *ZmLACS* genes within the same subgroup exhibited a high degree of homology in terms of type, number, and position. Moreover, the intron-exon structures of the *ZmLACS* gene family showed extensive similarity across subgroups. Overall, conserved motif and gene structure analyses of ZmLACSs suggested that members within subclades show conserved evolutionary relationships and may possess similar biological functions.

Gene duplication has long been recognized as a mechanism that amplifies gene families during evolution, serving as a potential source of evolutionary novelty [55]. Consistent with this, chromosomal localization analysis indicated that 11 *ZmLACS* genes were distributed across five chromosomes, suggesting that genetic variation plays a significant role in the evolution of maize *LACS* genes. The syntenic analysis further showed three colinear gene pairs in maize *LACS*, all arising from segmental duplication events, highlighting the fundamental role of these duplications in the evolution of *ZmLACS* genes. The Ka/Ks values of these three tandem duplication pairs were < 1, indicating strong negative selection driving the evolution of the *ZmLACS* gene family by removing deleterious variants. Interestingly, no colinear gene pairs were found between *Arabidopsis LACS* and maize *LACS*, suggesting a relatively distant genetic relationship between these species. In contrast, 15 orthologous gene pairs were identified by comparing the genome-wide data of pecan *LACSs* with *Arabidopsis*, highlighting a closer genetic relationship between pecan and Arabidopsis [56]. Our speculation is that the genetic relationship between maize and Arabidopsis is relatively distant. For example, maize is a typical monocot plant, while Arabidopsis belongs to a dicotyledonous plant. However, deeper insights into the molecular mechanisms underlying these evolutionary patterns necessitate further investigation.

*Cis*-acting regulatory elements play crucial roles as transcriptional gene regulatory units, influencing tissue-specific expression, and regulating responses to various stresses [57]. Herein, *cis*-elements responsible for tissue-specific expression, hormone responses, and responses to abiotic stress were comprehensively identified in the promoter regions of *ZmLACS* genes. Similar findings have been reported in several other species, such as apple, cotton, pecan, and wheat, reinforcing the understanding that *LACS* genes are involved in hormonal regulation, developmental processes, and responses to abiotic stresses in plants [41,56,58,59]. In addition, interestingly, the AT-rich element (binding site of AT-rich DNA binding protein (ATBP-1)) was only detected in the promoter region of *ZmLACS9.2*, and circadian (*cis*-acting regulatory element involved in circadian control) was specifically enriched in the promoter region of *ZmLACS8.2*. However, further extensive biochemical and genetic evidence is needed to deepen our understanding in this area.

Previous research has shown that LACS proteins activate long-chain FAs to generate lipid acyl-coenzyme A, thereby contributing to plant growth and development. In Arabidopsis, more than three LACS proteins have been shown to be involved in keratin and epidermal wax production [1,60]. Furthermore, the biochemical homeostasis of epidermal wax in Arabidopsis can enhance plant tolerance to water deficiency, salt stress, drought stress, and ABA treatment [61,62,63]. These studies strongly suggest that LACS proteins play a vital role in responding to environmental stress via regulating wax biosynthesis. In this study, we assessed the expression profiles of *ZmLACS* family genes across diverse tissues and under various treatment conditions. Comparison of *ZmLACS* expression levels across diverse tissues demonstrated that the majority of *ZmLACS* genes exhibited the highest expression in the meiotic tassel (V18). The high demand for LACS activities in floral tissues is linked to the strong expression of other lipid metabolic genes in flowers, indicating their involvement in active FA metabolism during meiotic tassel (V18) development [64,65]. Additionally, all *ZmLACS* genes showed elevated expression levels during seed development at 2, 4, and 8 DAP, suggesting their potential role in early FA accumulation in maize seeds. Moreover, the majority of *ZmLACS* genes exhibited significant responses to various stress treatments, including drought stress, salt stress, and ABA treatment. This implies that these genes play crucial roles in enhancing drought and salt stress resistance in maize. In addition, we also dissected the possible link between *cis*-element analysis and the expression profiles of *ZmLACS* family genes. Interestingly, no representative *cis*-element associated with specific tissues, such as seed and embryo, was detected on the promoter regions of *ZmLACS* family genes. Furthermore, with the exception of *ZmLACS8.2* and *ZmLACS9.2*, ABRE elements (*cis*-acting element involved in the abscisic acid responsiveness), closely related to drought stress, salt stress, and ABA treatment, were distributed on the promoter regions of the remaining *ZmLACSs*. As a consequence, these findings indicated that the data from promoter analysis may not provide significant evidence associated with expression patterns of *ZmLACS* genes. However, further extensive biochemical and genetic evidence is required to validate these hypotheses.

Notably, we should focus on two future research directions, such as investigating the regulatory networks of *ZmLACS* genes or conducting field trials to assess the environmental adaptability of maize with altered *LACS* gene expression. These research directions hold promise for advancing our understanding of plant biology and developing more sustainable agricultural practices. The identification and analysis of *LACS* gene families in different crops, such as wheat and cotton, have revealed their significant involvement in plant development and stress responses [41,59]. Therefore, further research into the regulatory networks of *LACS* genes could provide insights into the molecular mechanisms underlying plant growth, development, and stress responses, which could be leveraged for breeding more resilient and productive crop varieties. Furthermore, maize is one of the most important staple crops worldwide, and its yield and quality are influenced by a variety of agronomic traits [66]. Modern maize breeding has focused on improving these traits through selection for specific genetic markers associated with desirable phenotypes. By altering *LACS* gene expression, it may be possible to modify key agronomic traits such as yield, plant architecture, and stress tolerance. Field trials could help evaluate the performance of maize lines with modified *LACS* gene expression under different environmental conditions, providing valuable data for guiding future breeding strategies aimed at enhancing maize adaptability and productivity.

Here, we also pay attention to potential practical applications of our research. Interestingly, the theoretical foundation of maize LACSs provides a valuable starting point for engineering maize crops with improved stress tolerance or influencing agricultural practices. We speculate that, by leveraging genetic and molecular breeding approaches, pyramiding stress-tolerant alleles, and informed agricultural practices, it is possible to develop maize varieties that are better equipped to face the challenges posed by climate change and other environmental stresses [67,68].

In summary, our research comprehensively analyzed the *LACS* family genes in maize, analyzing their expression across diverse tissues and under various stress conditions. These findings significantly contribute to our understanding of the molecular mechanisms and regulatory networks governing *ZmLACS* genes, laying a theoretical groundwork for future genetic improvements.

## 5. Conclusions

Based on the maize genome, we identified 11 *ZmLACS* genes mapped to five chromosomes. Evolutionary and synteny analyses underscored the crucial role of segmental duplications in expanding the *ZmLACS* protein family. Examination of conserved motif composition and gene structures revealed shared features within distinct subclades. Analysis of *ZmLACS* promoter regions identified diverse *cis*-regulatory elements linked to tissue-specific expression, hormone responsiveness, and responses to abiotic stress. RT-qPCR experiments highlighted tissue-specific expression patterns and suggested roles in abiotic stress responses for *ZmLACS* genes. Finally, constructing a protein–protein interaction network of ZmLACS proteins provided insights into wax biosynthesis and regulatory mechanisms under abiotic stresses in maize. Altogether, these results provide a better understanding of the *ZmLACS* gene family and establish a theoretical foundation for further investigations aimed at enhancing maize productivity and stress resilience.

## Data Availability

The original contributions presented in the study are included in the article/Supplementary Materials, further inquiries can be directed to the corresponding author.

## Supplementary Materials

The following supporting information can be downloaded at: https://www.mdpi.com/article/10.3390/genes15080983/s1, Figure S1. Alignment of LACS proteins in maize and Arabidopsis. The AMP-binding domain is indicated by red box, while a conserved ACS signaling motif is indicated by blue box above the alignment. Figure S2. Detailed information of the AMP-binding domain and conserved ACS signaling motif in AtLACSs and ZmLACSs. Figure S3. Synteny assessment of LACS genes between maize and Arabidopsis. The collinear blocks within the genomes of both species are indicated by background gray lines. Figure S4. Ten conserved motifs of ZmCRF genes. Table S1. Primers used in this study. Table S2. Genome-wide characterization of the LACS proteins in maize. Table S3. Ka, Ks, and Ka/Ks values for duplication gene pairs of maize LACS genes. Table S4. Cis-acting elements analysis of LACS family members in mazie. Table S5. Detailed information on the interaction network of the ZmLACS family proteins.
