# Supplementary material for: Genome-Wide Identification and Characterization of Maize Long-Chain Acyl-CoA Synthetases and Their Expression Profiles in Different Tissues and in Response to Multiple Abiotic Stresses
Source: Genes (Basel). 2024 Jul 25;15(8):983. doi: 10.3390/genes15080983 (PMC11354158; doi:10.3390/genes15080983)
Supplement: Supplementary file 1 [file genes-15-00983-s001.zip › Supplementary data-LACS.pdf]

Genome-wide identification and characterization of maize long-chain acyl-CoA synthetases and their expression profiles in different tissues and in response to multiple abiotic stresses

Zhenwei Yan[1], Jing Hou[2], Bingying Leng[1], Guoqi Yao[1], Changle Ma[3], Yue Sun[4], Qian tong Liu[4], Fajun Zhang[1], Chunhua Mu[1], Xia Liu[1,*]

[1] Maize Research Institute, Shandong Academy of Agricultural Sciences, Jinan 250100, Shandong, China; Yanzwplant@sina.com (Z.Y.); Lengbingying@saas.ac.cn (B.L.); Yaoguoqi@ saas.ac.cn (G.Y.); Zhangfajun@saas.ac.cn (F.Z.)

[2] School of Agriculture, Ludong University, Yantai 264001, Shandong, China; houjing@m.ldu.edu.cn (J. H.)

[3] College of Life Sciences, Shandong Normal University, Jinan 250300, Shandong, China; machangle@sdnu.edu.cn (C.M.)

[4] College of Agronomy, Qingdao Agricultural University, Qingdao 266109, Shandong, China; sunyue3070601@163.com (Y.S.); 13356748152@163.com (Q.L.)

*Correspondences: Xia Liu (Liuxiamaize@163.com, Dr. Liu is responsible for the distributions of the material associated with this article)

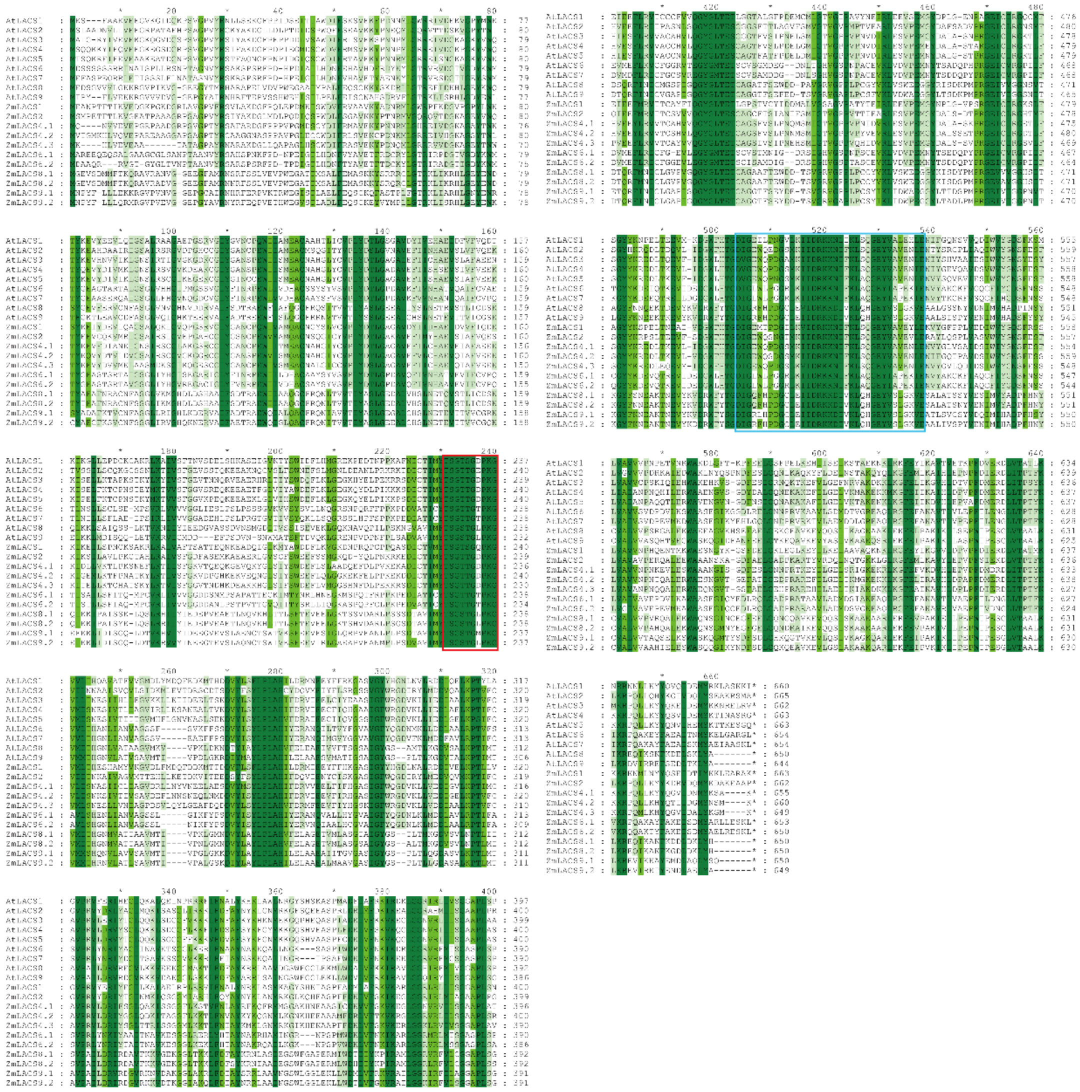

Figure S1. Alignment of LACS proteins in maize and Arabidopsis. The AMP-binding domain is indicated by red box, while a conserved ACS signaling motif is indicated by blue box above the alignment.

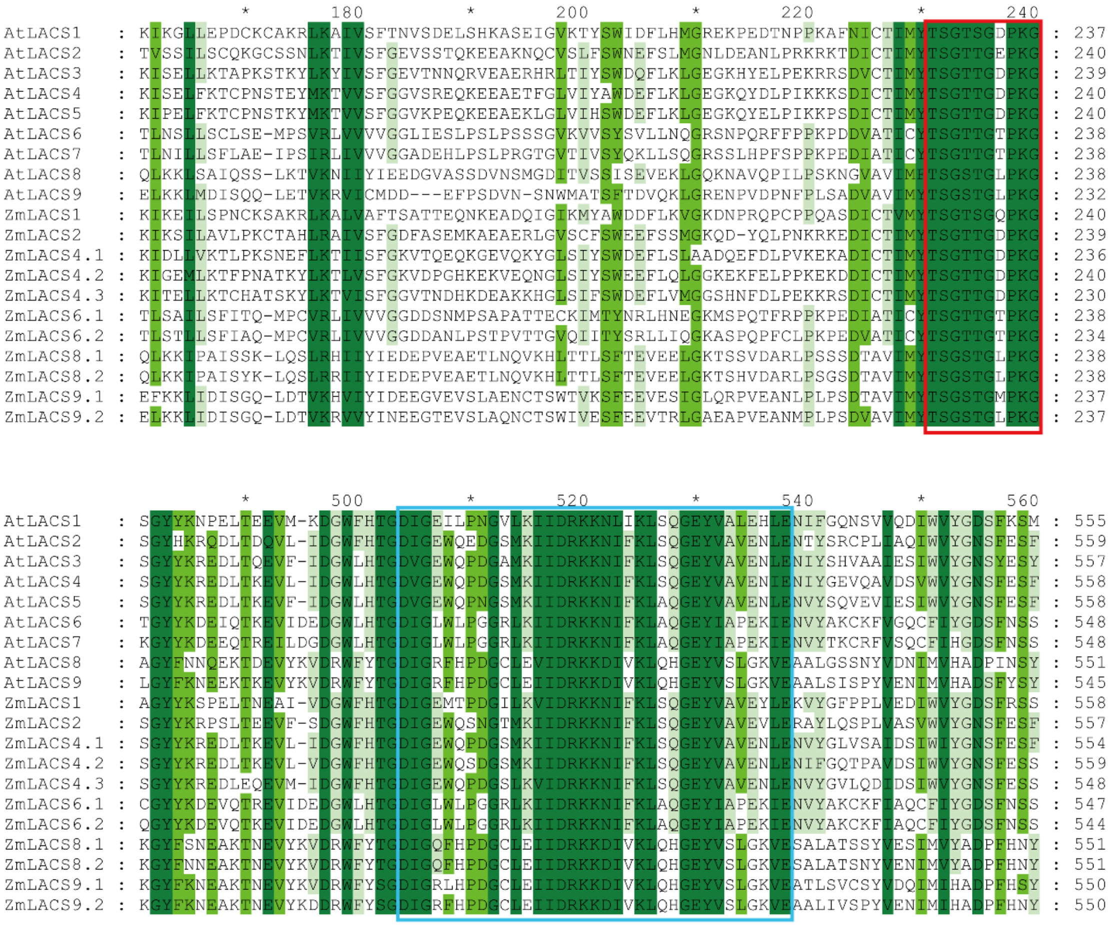

Figure S2. Detailed information of the AMP-binding domain and conserved ACS signaling motif in AtLACSs and ZmLACSs.

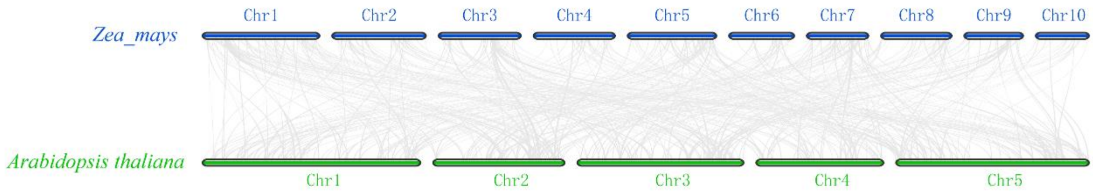

Figure S3. Synteny assessment of *LACS* genes between maize and Arabidopsis. The collinear blocks within the genomes of both species are indicated by background gray lines.

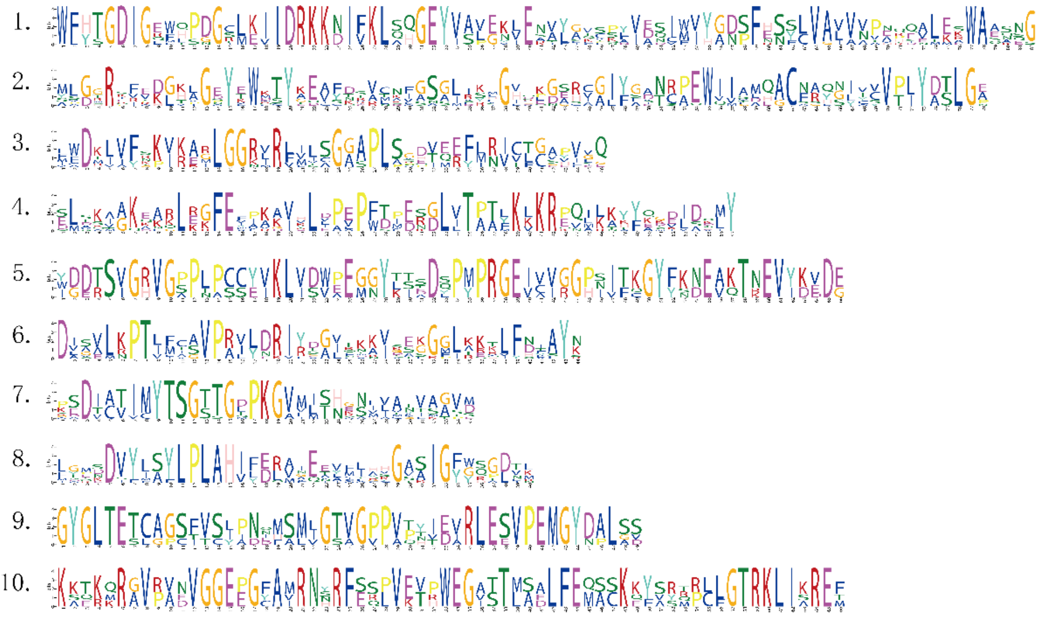

Figure S4. Ten conserved motifs of *ZmCRF* genes.
